# Selected inflammatory markers in uterine and peripheral blood of bitches with pyometra

**DOI:** 10.2478/jvetres-2026-0014

**Published:** 2026-03-12

**Authors:** Kinga Domrazek, Szymon Kanafa, Magdalena Krzeszewska Zachary, Roman Dąbrowski, Lourdes Soler, Piotr Jurka

**Affiliations:** Department of Small Animal Diseases and Clinic Warsaw,; Department of Pathology and Veterinary Diagnostics, Institute of Veterinary Medicine, Warsaw University of Life Sciences, 02-776 Warsaw, Poland; Department of Animal Reproduction, Faculty of Veterinary Medicine, University of Life Sciences in Lublin, 20-612 Lublin, Poland; Department de Bioquímica y Biología Molecular y Celular, Facultad de Ciencias, Universidad de Zaragoza, 50013 Zaragoza, Spain

**Keywords:** bitch, interleukin, local and systemic inflammation, pyometra cervical patency

## Abstract

**Introduction:**

Pyometra is a common and potentially life-threatening uterine infection in bitches, with severe local and systemic inflammation as symptoms. Studies exist on inflammatory markers in peripheral blood (PB), but little is known about their expression in uterine blood (UB). This study aimed to compare their concentrations simultaneously in UB and PB of bitches with pyometra, and to evaluate their diagnostic and prognostic potential.

**Material and Methods:**

Twenty-two bitches with confirmed pyometra (13 closed-cervix and 9 open-cervix cases) and six healthy controls were enrolled. Blood samples were collected from the cephalic and uterine veins during ovariohysterectomy. The concentrations of IL-1α, IL-1β, IL-6 and IL-10 were measured using an ELISA validated for dogs, and C-reactive protein (CRP) was measured by an immunoturbidimetric assay. These five inflammatory markers were compared statistically between the three groups of dogs and two types of blood sample using the Mann–Whitney U and Wilcoxon tests.

**Results:**

All markers were significantly elevated in affected bitches’ UB and PB. The IL-6 concentrations were significantly higher in UB than in PB, suggesting local production of this cytokine. Conversely, the IL-10 levels were higher in PB, possibly reflecting a systemic regulatory response. The concentrations of IL-1α and IL-1β differed insignificantly from each other. Those of CRP were significantly higher in open-cervix than in closed-cervix pyometra, indicating a stronger acute-phase response.

**Conclusion:**

The results assign IL-6 and CRP a potential role as biomarkers for the diagnosis and prognosis of pyometra. Simultaneous analysis of uterine and systemic inflammatory responses provides insight into the disease’s pathophysiology and may support more targeted therapeutic approaches.

## Introduction

Pyometra is an acute or chronic bacterial infection of the uterus characterised by the accumulation of inflammatory exudate in the uterine lumen, leading to both local and systemic clinical manifestations. The pathogenesis of pyometra remains incompletely understood, but it is suspected to be influenced by hormonal and bacterial factors. During the luteal phase, the uterine environment becomes conducive to microbial growth (15). Progesterone promotes endometrial gland proliferation, increased secretion, cervical closure and suppression of myometrial contractions while diminishing local leukocyte responses and uterine resistance to bacterial invasion. Although circulating levels of oestrogen and progesterone are typically not elevated in pyometra, the increased number and sensitivity of hormone receptors may amplify hormonal effects (5, 6, 15). Concurrent *corpora lutea* and follicular cysts are often observed in bitches with pyometra, further supporting a synergistic hormonal influence.

The most frequently isolated pathogen in pyometra cases is *Escherichia coli*; however, other bacteria may also be involved (34, 35). These bacteria and especially their endotoxins cause serious local and systemic inflammation. Pyometra is often associated with endotoxaemia, bacteraemia and disseminated infections, which may affect various organs and lead to lifethreatening complications (14).

Interleukins are inflammatory markers and reflect local inflammation, representing the immune system’s primary response to noxious insults (27). Interleukin 6 is a glycoprotein with a molecular weight of 26 kDa produced primarily by monocytes and macrophages (1, 20). Because of its broad spectrum of biological activity, it was historically referred to as the hepatocytestimulating factor, acute-phase response inducer or even as a hybrid factor (10). This cytokine mediates inflammation and the immune system’s broader initial response to harmful stimuli (27). Its main function is the regulation of the acute-phase response, largely through the stimulation of acute-phase protein production (27). The biological effects of IL-6 are exerted by its binding to two receptor types: a membrane-bound receptor (IL-6R) with relatively low affinity and a soluble receptor (sIL-6R). Both forms signal through a shared transducing component, gp130, which leads to downstream cellular activation and gene expression (1, 19). Interleukin 6 is rapidly and transiently produced in response to infections and tissue injury, contributing to host defence by enhancing acute-phase responses, haematopoiesis and immune functions. Its expression is tightly controlled at the transcriptional and post-transcriptional levels, ensuring that activation is swift but short-lived under normal conditions (31). Blocking IL-6 signalling is a therapeutic strategy commonly used to reduce the impact of autoimmune diseases (20). Its biological activity is closely associated with B lymphocytes, particularly in stimulating their final differentiation into plasma cells, which are essential for antibody production. This interleukin modulates the levels of transferrin, ceruloplasmin and haptoglobin, thus playing a role in maintaining iron and copper homeostasis in the body (27). However, when IL-6 production becomes persistently dysregulated, it may lead to chronic inflammation and contribute to the development and progression of autoimmune diseases. It therefore has dual roles as both a protective and pathogenic factor (13).

Interleukin 1 is a key mediator of the innate immune response and a central regulator of inflammation. It comprises two major isoforms, IL-1α and IL-1β, both of which are widely expressed in the body and particularly abundant in macrophages within lymphoid organs, as well as to some extent in endometrial tissue (17). The commonly used term IL-1 refers to its two forms: a and β. It is secreted by monocytes and macrophages and exhibits pleiotropic effects in modulating the inflammatory response. The proIL-1α form is intracellularly active, capable of binding nuclear DNA and can be released into the extracellular space (11). In contrast, proIL-1β requires cleavage by the cysteine protease IL-1β-converting enzyme to become biologically active as IL-1β (11). Once activated, IL-1 plays a crucial role in orchestrating the body’s defence mechanisms. Its biological activity is manifested through stimulation of lymphocyte activation, the production of acute-phase proteins and the synthesis of prostaglandins (11, 32). The cytokine mediates its effects *via* the IL-1 receptor type I (IL-1RI), leading to the activation of intracellular signalling pathways including the cyclic adenosine monophosphate response element–binding protein and NF-ĸB (15). Additionally, IL-1β interacts with IL-1 receptor type II and the IL-1 receptor accessory protein, contributing to the development of both local and systemic inflammatory responses (29).

During the early stages of inflammation, granulocytes are the predominant effector cells, while in chronic inflammation, mononuclear cells such as monocytes and macrophages become more prevalent. Both IL-1 and IL-6 influence the hypothalamic thermoregulatory centre, leading to an increase in body temperature (1). In addition to its pro-inflammatory role in injured tissues, IL-1 contributes to maintaining homeostasis across various systems by regulating immune and cellular responses. However, persistent overproduction or dysregulation of IL-1 signalling can lead to chronic inflammation and is implicated in the pathogenesis of several autoimmune and inflammatory diseases (17).

Interleukin-10, in contrast to the aforementioned mediators of the inflammatory response, plays an inhibitory role in the body’s immune response. It is produced by monocytes, macrophages and T lymphocytes (Treg – Tr1 and Th2 cells). Two receptors for IL-10 have been described: IL-10R1 and IL-10R2. Expression of these IL-10 receptors has been observed mainly in immune cells, including CD4^+^ and CD^+^ Tlymphocytes, B cells, dendritic cells, macrophages and natural killer cells (36). The pleiotropic effects of IL-10 lead to the inhibition of other cytokine production, suppression of the oxidative burst and reduction of class II major histocompatibility complex molecule expression on monocytes, thereby weakening the inflammatory response. Interleukin 10 is also attributed with a stimulatory role in the production of the interleukin-1 receptor antagonist (8, 16).

C-reactive protein (CRP) is an acute-phase protein whose expression increases markedly during infection or inflammation; serum concentrations commonly rise up to approximately 100-fold, whereas in severe systemic infections the increase may approach 1,000-fold compared with baseline (22). It is primarily produced by hepatocytes in the liver, but also by smooth muscle cells, macrophages, endothelial cells, lymphocytes and adipocytes (30). Morphologically, CRP consists of five identical subunits forming a cyclic pentamer and exists in two conformations – the native pentameric form (nCRP) and a modified monomeric form (mCRP) (30). In the circulation, CRP is present predominantly as nCRP, which is the isoform routinely quantified in standard serum assays. In contrast, mCRP is thought to be generated mainly at sites of inflammation through dissociation of the pentamer on activated cell membranes and microvesicles, and it is generally associated with distinct, often more pro-inflammatory biological effects. Therefore, increases in serum CRP primarily reflect systemic acute-phase upregulation of circulating nCRP, whereas isoform-related processes are expected to modulate local inflammatory mechanisms rather than determine baseline reference concentrations. These isoforms differ in their biological activity and receptor interactions, but the native form modulates inflammation by promoting the precipitation and agglutination of microorganisms, enhancing phagocytosis by binding cellular debris and activating the classical complement pathway (33). They also influence cytokine production by monocytes and macrophages. Although CRP levels are elevated in infections and inflammatory diseases, the role of its isoforms in the development and progression of these conditions remains poorly understood (30). In healthy dogs, serum CRP concentrations are low and typically remain below the assay-specific reference limit (12); however, it is important to note that the values established as physiological for CRP are different for each analytical method and type of assay. For instance, Otabe *et al*. (23) reported serum CRP concentrations in healthy dogs ranging from 0.8 to 22.6 μg/mL. During active inflammation, serum CRP concentrations most commonly increase up to approximately 100-fold relative to baseline (12). The magnitude of this increase depends on the severity and systemic extent of the inflammatory response, and in severe systemic infections it may be substantially higher (22). In female dogs which have undergone ovariohysterectomy, CRP levels rise rapidly post-surgery and then decline significantly after a few days in a dynamic response to tissue injury (12).

While inflammatory markers such as IL-6, IL-1α, IL-1β and CRP have been studied in systemic infections, their specific expression in the uterine (UB) and peripheral blood (PB) of bitches with pyometra remains poorly understood. Investigating these markers may provide insights into the local and systemic inflammatory response associated with pyometra, potentially guiding future diagnostic and therapeutic approaches. This study aims to investigate the expression of selected inflammatory markers, specifically interleukins (IL-6, IL-1α and IL-1β) and the acute-phase protein CRP, in the UB and PB of bitches diagnosed with pyometra.

## Material and Methods

### Experimental design and ethics approval

There was a total of 22 animals in the experimental groups in which pyometra was confirmed by clinical examination and additional tests. Thirteen of them were individuals with confirmed closed-cervix pyometra (CCP) and nine were bitches with open-cervix pyometra (OCP). Dogs with confirmed pyometra had an average age of approximately 7.6 years and an average body weight of 16.1 kg. The dogs in the control group of six animals had an average age of 1.2 years and an average body weight of 20.8 kg. Some of the dogs were brought by their owners for surgery, and some came from a shelter. The study did not require specific ethical approval. The animals were patients being treated according to the ethical standards set down in national legislation. The owner’s agreement was obtained before all procedures According to current regulations, no ethics committee approval is needed for routine veterinary procedures.

Pyometra was diagnosed based on the medical history, clinical symptoms (anorexia, polydipsia, polyuria and ataxia), a cytology swab and abdominal ultrasonography performed using a SonoScape S9 ultrasound system equipped with a microconvex probe (Guangdong, China). Each dog underwent a comprehensive clinical examination to rule out other abnormalities or diseases. None of the dogs were pregnant or receiving contraceptives at the time of the study. Dogs diagnosed with pyometra were further classified into two groups, OCP and CCP, based on the presence of purulent vaginal discharge, confirmed by cytological examination revealing a field filled with degenerative neutrophils and bacteria. Blood tests (morphology and biochemistry parameters) were performed for each bitch.

Blood samples were collected during ovariohysterectomy surgery in both the OCP and CCP groups from the cephalic vein (representing peripheral circulation) and the uterine vein (representing local circulation) into 2 mL tubes intended for biochemical blood tests; CCP dogs were clinically healthy bitches presented for routine elective spaying, and the blood sampling procedure was performed identically in the study and control groups. The samples were centrifuged (MPW223e; MPW Med Instruments, Warsaw, Poland) at 4,000 rpm for 5 min, and the serum was separated and stored at –71°C until analysis. Only serum without excess lipids was used.

### Laboratory test

Interleukins were measured using two ELISA sandwich tests (Acuvet Biotech, Zaragoza, Spain) that had previously been developed and validated (24). A canine-specific turbidimetric immunoassay (Turbovet canine CRP, Acuvet Biotech) was used to determine CRP. The test was performed according to the procedure described by Pineiro *et al*. (24).

### Statistical analysis

All tested parameter data were summarised with their median and range. The comparison between two groups was made using the Mann–Whitney U test and between two groups corresponding to the same animal but different sources of blood using the Wilcoxon matched-pairs signed-rank test. The effectiveness of pairing was tested using Spearman’s rank correlation coefficient. Statistical significance was set at 0.05 (P-value < 0.05) and was considered statistically significant. Statistical analysis was performed with GraphPad Prism software (GraphPad, Boston, MA, USA).

## Results

### Morphology and biochemistry of the blood

Peripheral blood count analysis showed a significant increase in WBC in the samples from OCP and CCP dogs compared to control dogs. The medians were 21.43, 19.87 and 7.35 (×10^9^/L) respectively. No significant differences were found in the comparison of WBC in samples from bitches with one type of uterine inflammation with the samples from bitches with the other. Similarly, no differences were found in RBC, HCT, HGB or PLT (Table 1).

**Table 1.** Medians of morphological and biochemical parameters measured in peripheral blood with the minimum–maximum range

	OCP	CCP	C	Pair-wise comparison	P-value
WBC (×10^9^/L)	21.43 (16.76–24.9)	19.87 (14.76–24.53)	7.35 (6.7–8.4)	C *vs* OCP	0.0007
				C *vs* CCP	0.0001
				CCP *vs* OCP	0.3431
RBC (×10^12^/L)	4.9 (4.6–6.1)	5.2 (3.6–6.6)	4.8 (4.3–5.8)	CCP *vs* OCP	0.4875
				C *vs* CCP	0.5369
				CCP vs OCP	0.8349
HCT (%)	45 (31–54)	43 (28–59)	45 (33–50)	C *vs* OCP	0.9873
				C *vs* CCP	0.8349
				CCP *vs* OCP	0.983
HGB (g/dL)	17.2 (14.7–25.8)	17.89 (13.7–20.3)	17.9 (14.9–20.3)	C *vs* OCP	0.8765
				C *vs* CCP	0.7659
				CCP *vs* OCP	0.6919
ALT (IU)	51.5 (29–87)	46.5 (19–95)	26 (12–31)	C *vs* OCP	0.0023
				C *vs* CCP	0.0075
				CCP *vs* OCP	0.5073
AST (IU)	45 (12–54)	34 (12–64)	27.5 (18–35)	C *vs* OCP	0.0989
				C *vs* CCP	0.3916
				CCP *vs* OCP	0.137
ALP (IU)	82.5 (45–99)	93.5 (23–152)	139.5 (129–152)	C *vs* OCP	0.0007
				C *vs* CCP	0.0023
				CCP *vs* OCP	0.4137
CR (mg/dL)	1.7 (1.4–1.9)	1.7 (0.5–2)	0.95 (0.7–1.2)	C *vs* OCP	0.0007
				C *vs* CCP	0.0486
				CCP *vs* OCP	0.5045
Urea (mg/dL)	33 (19–50)	45.5 (21–59)	21 (13–41)	C *vs* OCP	0.1668
				C *vs* CCP	0.0014
				CCP *vs* OCP	0.0358
PLT (10^9^/L)	361.5 (234–643)	291 (194–498)	405 (231–543)	C *vs* OCP	0.7546
				C *vs* CCP	0.1712
				CCP *vs* OCP	0.3144

1 OCP – open-cervix pyometra group; CCP – closed-cervix pyometra group; C – healthy control group. Statistical significance was established at P ≤ 0.05

Differences were found in blood biochemistry. Alanine aminotransferase showed higher activity in the blood of animals suffering from OCP and CCP compared to the blood of unaffected animals: 51.5, 46.5 and 26 IU, respectively. A statistically significant difference was also found in the activity of ALP. Here, the situation was the opposite to that of ALT, because the activity was lower in the blood taken from OCP and CCP bitches than in the blood from controls. The medians were 82.5, 93.5 and 139.5 IU, respectively (Table 1). Statistical differences were demonstrated for CR and urea. The CR concentration was 1.7 mg/dL in OCP and CCP and exceeded the control median concentration of 0.95 mg/dL. The urea concentrations differed between CCP and control, being 45.5 and 21 mg/dL, respectively. Also, comparing urea concentrations between types of inflammation, a statistically significant difference was observed between that of CCP and the 33 mg/dL of OCP. The CR concentrations measured in blood from OCP and control animals did not show any significant differences (Table 1). Neither were differences demonstrated in AST activity between any study groups.

**Fig. 1.**
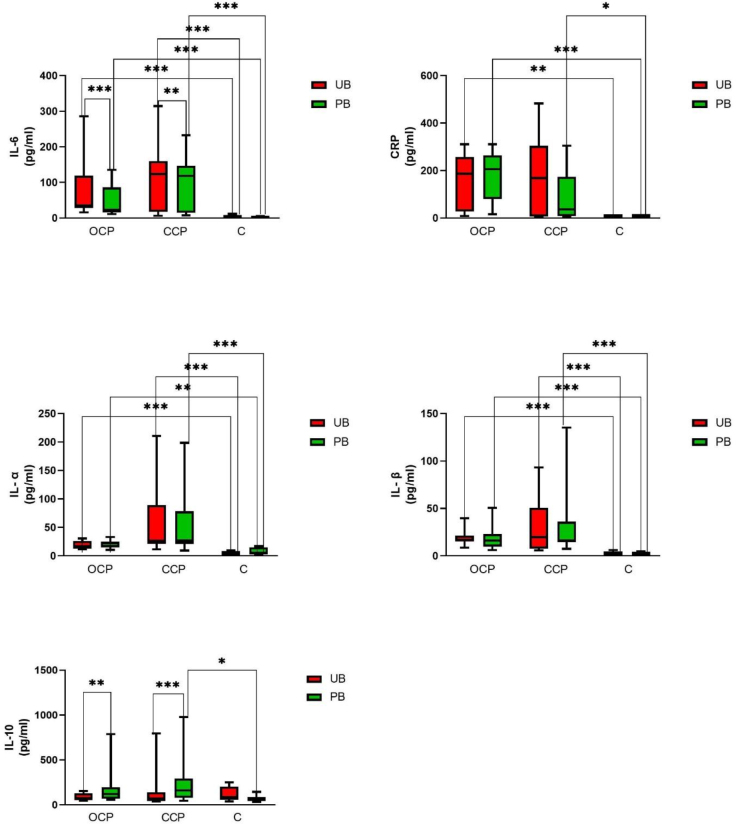
Medians of the studied parameters from peripheral blood (PB) and uterine blood (UB) along with the minimum-maximum range). OCP – open-cervix pyometra group; CCP – closed-cervix pyometra group. Statistical significance was established at P ≤ 0.05 and marked with *

Table 2 presents the median values of the studied biomarkers in PB and UB by type of pyometra: OCP, CCP and none (control animals). In the cases of CRP, IL-1α and β and IL-6, statistically significant differences were shown between their concentrations in PB and UB from diseased dogs compared to these concentrations in PB and UB from healthy dogs (Fig. 1). During inflammation, the concentrations of these components increased, and this occurred in both open and closed pyometra. Only the CRP concentration measured in UB drawn from CCP bitches was insignificant in its difference from the concentration in blood drawn from healthy bitches, and this difference failed significance by only a small margin (P = 0.06).

**Table 2.** Medians of the studied parameters from peripheral blood (PB) and uterine blood (UB) along with the minimum–maximum range (pg/mL)

		OCP	CCP	C		P-value
CRP	PB	205.8 (16.1 – 310.9)	36.15 (6.8–304.3)	7.8 (6.8–10.6)	OCP *vs* C	0.0007
					CCP *vs* C	0.0336
					OCP vs CCP	0.0299
	UB	186.8 (8–310.9)	168.4 (5–482.5)	6.6 (5.1–10.1)	CCP *vs* C	0.0013
					CCP vs C	0.0644
					OCP *vs* CCP	0.6627
	P-value	0.5781	0.0728			
IL-1α	PB	22.16 (10.5–33.24)	26.3 (11.37–210.5)	5.46 (2.2–16.99)	OCP vs C	0.008
					CCP *vs* C	0.0004
					OCP *vs* CCP	0.1147
	UB	16.91 (11.4–30.57)	27.03 (9.3–198.7)	3.015 (0–9.6)	OCP *vs* C	0.0007
					CCP *vs* C	0.0001
					OCP *vs* CCP	0.0658
	P-value	0.3828	0.9526			
IL-1β	PB	16.2 (6.21–50.62)	16.17 (7.4–135.2)	0.215 (0–4.85)	OCP *vs* C	0.0007
					CCP *vs* C	0.0001
					OCP *vs* CCP	0.5582
	UB	16.92 (8.59–39.56)	19.66 (5.87–93.15)	1.15 (0–6.04)	OCP *vs* C	0.0007
					CCP vs C	0.0001
					OCP vs CCP	0.0658
	P-value	0.4609	0.9697			
IL-6	PB	23.55 (11.18–135.1)	118.2 (7.39–232.5)	0 (0–5.19)	OCP *vs* C	0.0007
					CCP *vs* C	0.0001
					OCP *vs* CCP	0.2083
	UB	35.93 (15.99–285.4)	123.6 (6.39–314.5)	3.195 (0–11.58)	OCP *vs* C	0.0007
					CCP *vs* C	0.0005
					OCP *vs* CCP	0.6378
	P-value	0.0078	0.001			
IL-10	PB	123 (56.71–789)	161.5 (45.1–979.6)	60.38 (31.05–145.3)	OCP *vs* C	0.0636
					CCP *vs* C	0.0126
					OCP *vs* CCP	0.6101
	UB	76.25 (45.71–154.9)	71.98 (37.77–795.8)	89.08 (39.6–253.4)	OCP *vs* C	0.5728
					CCP *vs* C	0.4936
					OCP *vs* CCP	0.8076
	P-value	0.0078	0.0005			

1 CRP – C-reactive protein; OCP – open-cervix pyometra group; CCP – closed-cervix pyometra group; C – healthy control group. Statistical significance was established at P ≤ 0.05

In the comparison of biomarkers in same-source blood samples by cervix patency status, a significant difference was observed only in CRP measurements in PB, where the median concentration in OCP was 205.8 pg/mL and in CCP was only 36.15 pg/mL. Only in one other biomarker was a concentration difference close to significance (P-value = 0.0658) measured: in IL-1α, where the median concentration was higher in the CCP group than in the OCP group when examining UB (Table 2, Fig. 1).

An interesting observation is that the concentration of IL-10 in UB did not differ between the OCP, CCP and control groups; however, a statistically significant difference between its concentrations in PB in CCP and its concentration in PB in controls (P-value = 0.0126), and one close to significance between the concentration in PB in OCP and the corresponding one in controls (P-value = 0.0636) were noted. In both cases, the concentration of IL-10 was higher than in the control during inflammation: the median was 123 pg/mL in OCP and 161.5 pg/mL in CCP (Table 2, Fig. 1)

Comparisons of the same biomarker concentrations in PB and in UB were also made. These showed that the concentration of IL-6 was statistically significantly higher in UB regardless of cervical patency status. A statistically significant difference was also observed in the case of IL-10, but here its higher concentrations were noted in PB. The test of the remaining parameters did not return any differences of significance (Table 2, Fig. 1).

## Discussion

For the first time, to the best of our knowledge, the levels of the pro-inflammatory cytokines IL-6, IL-1α and IL-1β were simultaneously compared in both peripheral and uterine blood of bitches diagnosed with pyometra in the present study. Previous research has primarily focused on analysing these markers in serum, and has not made extensive data available regarding their concentrations in the local uterine environment (27). The results obtained in this study provide new insights into both systemic and local inflammatory responses during the course of pyometra and may serve as a foundation for future research on potential diagnostic and prognostic biomarkers for this condition in dogs.

The present findings revealed statistically significant differences in the concentrations of IL-6, IL-1α, IL-1β and CRP between bitches with pyometra and the healthy control group, in both PB and UB samples. These results confirm that pyometra elicits a strong inflammatory response not only systemically but also locally within the uterus. Sasidharan *et al*. (27) discovered pronounced upregulation of IL6 expression in the endometrium and marked increases of serum IL6 concentrations in all grades of pyometra. Other authors found evaluated levels of CRP (4) and interleukins in bitches with pyometra (3). However, all of those results were based on peripheral and not uterine blood.

An important observation from this study is the significantly higher concentration of IL-6 in uterine blood than in peripheral blood in both open and closed pyometra. This finding strongly suggests local production of IL-6 at the site of infection. Some authors have suggested that this cytokine is one that can act in both pro-inflammatory and anti-inflammatory ways, depending on the nature of the inflammation. It is produced by several types of cells, including macrophages, which release IL-6 when they are activated by antigens (2).

Macrophage production may explain the UB content of IL-6 to some extent, because this would be mediated by a localised microbial presence. Pyometra is a polyaetiological disease in which bacterial antigens are also involved. Lopes *et al*. (21) found thirty different bacterial isolates in cultures of intrauterine content from bitches and queens suffering pyometra. *Escherichia coli* was isolated from 57.7% of samples and correlated highly with severe endometrial lesions. These lesions had marked content of mixed inflammatory infiltrate (lymphocytes, plasma cells, macrophages and neutrophils) and, in glands, were associated with necrosis (21). Those findings may confirm our observations, as macrophages present in severe endometrial lesions could be a source of IL-6 in uterine blood.

In contrast to IL-6, IL-10 was significantly more concentrated in peripheral blood than in uterine blood. This pattern may indicate a systemic mechanism for regulating and limiting the inflammatory response (28). Interleukin 10 is known for its anti-inflammatory properties, including the ability to inhibit the production of pro-inflammatory cytokines (31). The increase in IL-10 levels in the peripheral circulation, as opposed to its modulation in the endometrium, has also been confirmed by other authors (18, 26). Its elevated levels in the systemic circulation in pyometra could reflect the body’s attempt to counterbalance the local inflammation and prevent excessive immune activation that could lead to systemic damage.

Despite IL-1α and IL-1β being produced locally by activated macrophages and epithelial cells at sites of inflammation as stated by Pyrillou *et al*. (25), their concentrations in UB did not significantly exceed those in PB. This may be because of the rapid diffusion of these cytokines into systemic circulation following their release, leading to comparable levels in both compartments. Additionally, IL-1β in particular requires activation *via* the inflammasome and caspase-1, which might be tightly regulated to prevent excessive local damage (11). It is also possible that IL-1 family cytokines, once secreted, bind rapidly to local receptors or are neutralised by soluble receptors and inhibitors, limiting their accumulation in the local bloodstream (9). These mechanisms could collectively contribute to the observed lack of strong uterine–peripheral gradients for IL-1α and IL-1β, despite their known local expression.

In turn, the lack of differences in CRP concentrations between uterine blood and peripheral is probably due to the location of synthesis of this protein being the liver, from where it spreads through the body and reaches the uterine vein (30) and the cephalic vein. On the other hand, CRP concentrations were significantly higher in bitches with open-cervix pyometra compared to those with closed-cervix pyometra. These results are contradictory to those of Dąbrowski *et al*. (7), who besides comparing CRP in the two cervical patency statuses also compared it in UB and PB. More research on bigger groups is needed to elucidate CRP expression mechanisms in canine pyometra.

The results of this study suggest that the analysed inflammatory markers, particularly IL-6 and CRP, but also IL-1α and IL-1β, may be useful markers for the early detection of and assessment of the severity of inflammation in pyometra. Their significantly elevated concentrations in affected dogs, in both local and systemic compartments, reflected active immune responses that correlated with disease presence. Interleukin 6 and CRP appear especially promising as diagnostic and prognostic tools because of their strong expression and measurable dynamics. While IL-1α and IL-1β were also consistently elevated, their lack of variation between compartments or disease forms suggests a more general role in the inflammatory cascade. Interpreted together, the measured parameters may aid in early diagnosis, evaluation of disease progression and monitoring of treatment effectiveness in clinical settings.

This study has several limitations. First, the sample size, particularly in the control group, was relatively small, which may weaken the statistical power and constrain the generalisability of the results. Secondly, the age differences between the groups (younger control animals *vs* older bitches with pyometra) could have influenced the levels of inflammatory markers, as immune system function and hormonal status vary with age. Thirdly, the study did not account for possible comorbidities or individual variability in immune responses which might have affected cytokine expression. Additionally, the cross-sectional design barred assessment of changes in inflammatory marker levels over time or in response to treatment. Finally, while uterine blood sampling is a valuable method, it may not fully capture local tissue-level cytokine dynamics or the exact cellular sources of the measured markers.

## Conclusion

This study confirms that IL-6, IL-1α, IL-1β, IL-10 and CRP are significantly elevated in bitches with pyometra, indicating both local and systemic inflammation. Particularly high levels of IL-6 were measured in uterine blood, suggesting local production of the cytokine, while increased IL-10 in peripheral blood may have reflected systemic immune regulation. Among the tested biomarkers, IL-6 and CRP appear most promising as potential diagnostic and prognostic tools. These findings provide new insight into the inflammatory profile of pyometra and may contribute to improved disease assessment, although further studies on larger cohorts are needed.
